# Modulatory Effects of Spectral Energy Contrasts on Lateral Inhibition in the Human Auditory Cortex: An MEG Study

**DOI:** 10.1371/journal.pone.0080899

**Published:** 2013-12-09

**Authors:** Alwina Stein, Alva Engell, Hidehiko Okamoto, Andreas Wollbrink, Pia Lau, Robert Wunderlich, Claudia Rudack, Christo Pantev

**Affiliations:** 1 Institute for Biomagnetism and Biosignalanalysis, University Hospital, Münster, Germany; 2 Department of Integrative Physiology, National Institute for Physiological Sciences, Okazaki, Japan; 3 Department of Otolaryngology, University Hospital, Münster, Germany; Osaka University Graduate School of Medicine, Japan

## Abstract

We investigated the modulation of lateral inhibition in the human auditory cortex by means of magnetoencephalography (MEG). In the first experiment, five acoustic masking stimuli (MS), consisting of noise passing through a digital notch filter which was centered at 1 kHz, were presented. The spectral energy contrasts of four MS were modified systematically by either amplifying or attenuating the edge-frequency bands around the notch (EFB) by 30 dB. Additionally, the width of EFB amplification/attenuation was varied (3/8 or 7/8 octave on each side of the notch). N1m and auditory steady state responses (ASSR), evoked by a test stimulus with a carrier frequency of 1 kHz, were evaluated. A consistent dependence of N1m responses upon the preceding MS was observed. The minimal N1m source strength was found in the narrowest amplified EFB condition, representing pronounced lateral inhibition of neurons with characteristic frequencies corresponding to the center frequency of the notch (NOTCH CF) in secondary auditory cortical areas. We tested in a second experiment whether an even narrower bandwidth of EFB amplification would result in further enhanced lateral inhibition of the NOTCH CF. Here three MS were presented, two of which were modified by amplifying 1/8 or 1/24 octave EFB width around the notch. We found that N1m responses were again significantly smaller in both amplified EFB conditions as compared to the NFN condition. To our knowledge, this is the first study demonstrating that the energy and width of the EFB around the notch modulate lateral inhibition in human secondary auditory cortical areas. Because it is assumed that chronic tinnitus is caused by a lack of lateral inhibition, these new insights could be used as a tool for further improvement of tinnitus treatments focusing on the lateral inhibition of neurons corresponding to the tinnitus frequency, such as the tailor-made notched music training.

## Introduction

The amplitude of the N1m response evoked by a pure tone stimulus is reduced when a preceding masking noise with a digital notch filter centered at the frequency of the pure tone is presented [1], [2], [3]. It was proposed that the mechanism underlying this finding was lateral inhibition evoked by spectral energy contrasts.

**Figure 1:**
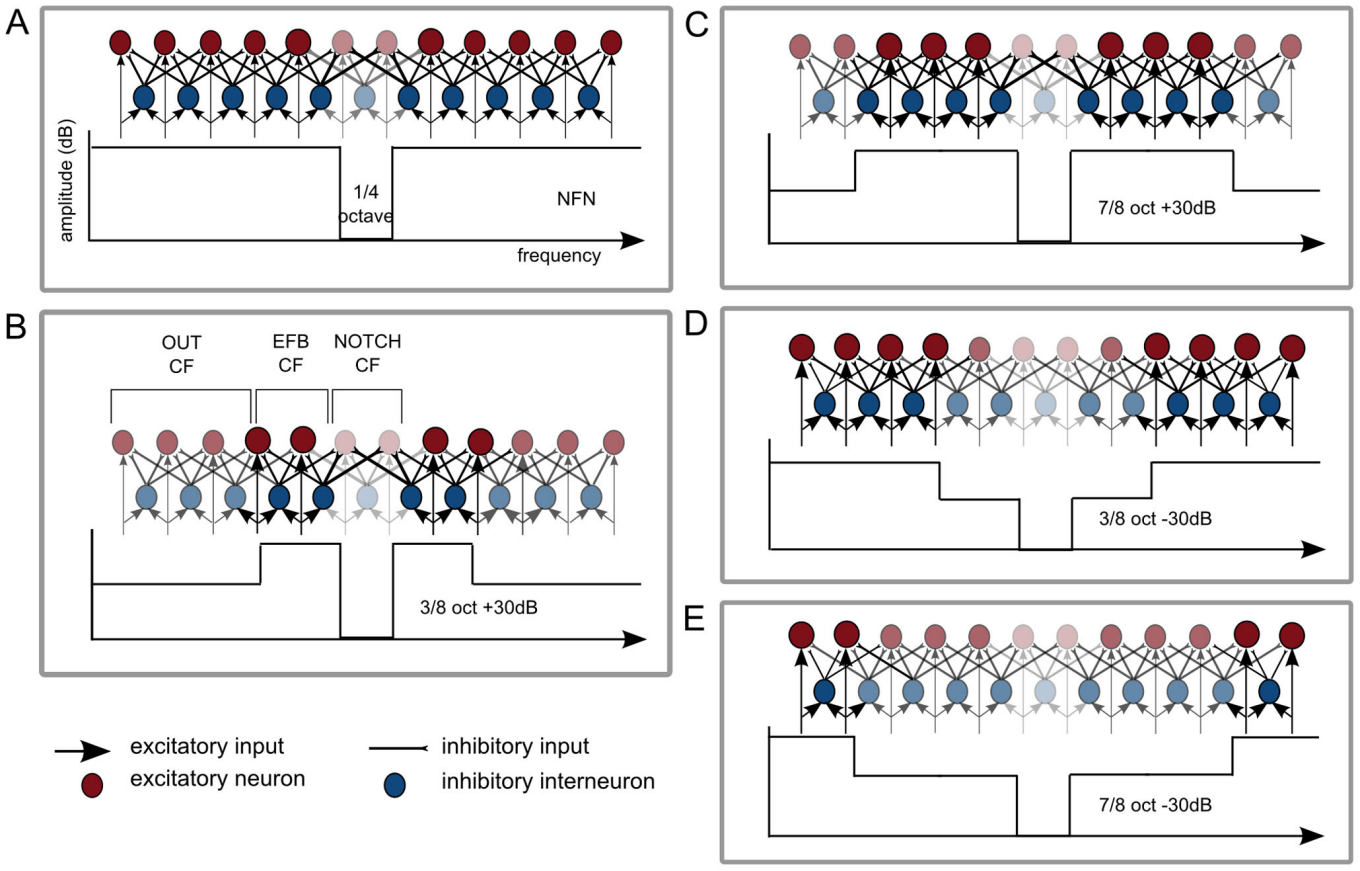
Schematic representation of neural mechanisms as evoked by the masking stimuli (MS). A. The lower part of the figure shows the frequency spectrum of the MS. The upper part is a highly simplified representation of neural activity in the auditory cortex as evoked by the MS. Neurons (excitatory = red, inhibitory = blue) are represented tonotopically. Transparent colors indicate reduced activation of inhibitory and excitatory neurons. Enlarged circles represent enhanced activation of neurons. B. Neurons with characteristic frequencies (CF) corresponding to the outer frequencies of the masking stimulus (OUT CF), the edge frequency bands (EFB) around the notch (EFB CF) and the frequencies within the notch (NOTCH CF) are further defined. A, B, C, D and E show the hypothesized modulation of neural mechanisms by MS with different spectral contrasts. The amount of lateral inhibition of neurons with NOTCH CF is hypothesized to be modulated in the following order (beginning with the greatest lateral inhibition of NOTCH CF): 3/8 oct +30 dB >7/8 oct +30 dB>NFN >3/8 oct −30 dB >7/8 oct −30 dB.

The auditory cortex is organized tonotopically [4], [5], [6]. When it receives afferent input from lower levels of the auditory system, excitatory neurons with the same characteristic frequencies (CF) are activated. Inhibitory interneurons also become excited and spread inhibition to adjacent neurons with lower or higher CF [7]. Because neurons with CF corresponding to frequencies within the notch (NOTCH CF) are not excited during stimulation with notch-filtered noise (NFN), the neurons with CF corresponding to the edge frequency bands around the notch (EFB) are not inhibited by collaterals of neurons with NOTCH CF. The result is an enhanced activation of neurons with CF corresponding to the EFB (EFB CF) and, consequently, a pronounced lateral inhibition of neurons with NOTCH CF (cf. Figure 1 A).

This hypothesis has been supported by varying the notch bandwidth [1] and notch position [2], revealing that the decrease in N1m response depends upon the distance between the excited EFB and the center frequency of the notch. The closer the EFB to the center frequency of the notch (though still not in a critical band range), the greater the N1m amplitude decrement. It might be argued that these effects could have been the result of peripheral masking, namely a forward masking effect in the cochlea. But since previous studies demonstrated that the forward masking effect disappears after 200 ms in the auditory nerve [8], [9] (for further arguments see also [10]) and the above mentioned studies found an N1m decrement after 500 ms [1], [2], [3], pure forward masking mechanisms in the auditory periphery cannot explain this effect. Instead, lateral inhibitory processes in central auditory structures seem to be the more plausible explanation.

A very recent study has also revealed that the edges of the notch have the greatest influence on the lateral suppression of neurons with NOTCH CF in the auditory cortex of guinea pigs [10]. This was shown by varying the notch bandwidth, transition steepness and depth, thereby varying the spectral energy contrast of NFN. It was furthermore shown that after listening to 3 hours of notched music on three consecutive days, the N1m response evoked by the frequency band within the notch decreased, while the N1m response evoked by frequencies far outside the notch was not affected [11]. These results again support the hypothesis that lateral inhibition is evoked by spectral energy contrasts in sound stimuli.

Lateral inhibition is an important basic neural mechanism in the sensory systems. In the case of the auditory modality, its functional role is, for example, the extraction of the spectral profiles of a sound [12], the enhancement of spectral information of a sound within surrounding noise [13] or the detection of frequency changes [14].

Indeed, a balance of neural excitation and inhibition is fundamental for an adequate sound processing. If this balance is disturbed, dysfunctions such as the perception of sounds without any external source can appear [15]. Noise-induced chronic tinnitus, for instance, seems to be caused by the reduction of lateral inhibition in the auditory system resulting in a reorganization of the auditory cortex [16]. This reorganization leads to an over-representation of some frequencies, which results in the perception of tinnitus [17], [18], [19]. Listening to music with a notch filter centered at the tinnitus frequency (tailor-made notched music training, TMNMT), however, resulted in a reduction of the neural activity evoked by the tinnitus frequency and in a reduction of the tinnitus loudness [20], [21]. Hence, mechanisms of lateral inhibition can be a cause of tinnitus perception, yet also a treatment strategy for relieving this perception.

Successfully identifying ways to modulate lateral inhibition could have an important impact not only on the functional investigation of the human auditory cortex, but also for enhancing treatments for chronic tonal tinnitus, such as TMNMT. Previous studies have investigated the modulation of lateral inhibition by introducing a notch filter and modifying its parameters [1], [2], [10]. Having identified the most effective notch parameters for the induction of lateral inhibition, however, this approach cannot be further expanded. The question therefore arises whether the manipulation of other parameters might enhance the effect of lateral inhibition.

## Objectives

The aim of this study was to investigate the influence of the EFB in NFN upon the lateral inhibition of neurons with NOTCH CF. We hypothesize that varying the energy in the EFB of NFN, while keeping the notch bandwidth and transition steepness constant, will result in a modulation of the lateral inhibition of neurons with NOTCH CF.

It is known that the N1m amplitude increases with increasing sound intensity [22], [23]. This phenomenon was reported by these authors to be the result of greater activation of neurons with CF corresponding to the frequency of the tonal stimulus. It was also shown by means of functional magnetic resonance imaging (fMRI) measurements that an increased sound level also led to increased negative blood oxygenation level dependent responses, which was interpreted as implying increased lateral inhibition of the adjacent non-excited neurons [24].

When amplifying the EFB, the corresponding neurons are most strongly activated, in comparison to neurons with other CF, because of the greater afferent excitatory input they have received. In addition, they receive only weak lateral inhibition from neighboring neurons, since neighboring neurons receive either less afferent excitatory input (neurons with CF outside the notch and EFB; OUT CF) or no afferent excitatory input at all (neurons with NOTCH CF). We therefore assume that neurons with EFB CF should laterally inhibit their neighboring neurons to the greatest extent, when NFN with amplified EFB is presented as compared to NFN without any additional spectral contrasts (cf. Figure 1 A–C).

To investigate the influence of the EFB in NFN systematically, an attenuation of the EFB should also be considered. When attenuating the EFB, neurons with OUT CF receive the strongest afferent excitatory input. Neurons with EFB CF receive less afferent input and are additionally inhibited by collaterals of neurons with OUT CF. Therefore neurons with NOTCH CF should be less inhibited by collaterals of neurons with EFB CF when presenting NFN with attenuated EFB as compared to NFN with no additional spectral energy contrasts (cf. Figure 1 A, D, E).

We furthermore hypothesize that the width of EFB amplification or attenuation also has an impact on lateral inhibition. The effect of lateral inhibition is known to have an influence on neurons with CF up to two octaves away from the CF of the excited neurons [25]. However, excited neurons inhibit adjacent neurons more strongly than more distant neighbors [26]. When amplifying a narrow width of EFB, neurons with OUT CF are closer to neurons with CF corresponding to frequencies near to the notch edges. Because neurons with OUT CF are activated to a lesser extent, as compared to neurons with EFB CF, and therefore have a smaller lateral inhibitory influence on EFB CF, the EFB CF should be even more strongly activated and consequently laterally inhibit the NOTCH CF more strongly than a broader width of EFB amplification (cf. Figure 1 B and C).

On the other hand, when attenuating EFB, neurons with OUT CF have the greatest lateral inhibitory impact on neurons with NOTCH CF (as described above). A narrower bandwidth of EFB attenuation has a smaller distance between neurons with OUT CF and neurons with NOTCH CF than a broader bandwidth of attenuation, with the result that greater lateral inhibition of NOTCH CF occurs (cf. Figure 1 D and E).

Two hypotheses were tested:

1. NFN with amplified EFB induces greater lateral inhibition of neurons with NOTCH CF than NFN without additional spectral energy contrasts. NFN with attenuated EFB induces lower levels of lateral inhibition of neurons with NOTCH CF than NFN without additional spectral energy contrasts.
2. A steady decrement of lateral inhibition of neurons with NOTCH CF is induced by the various MS in the following order (beginning with the greatest lateral inhibitory effect):

Narrow EFB amplification>broad EFB amplification>NFN (with no additional spectral energy contrasts)>narrow EFB attenuation>broad EFB attenuation.

These hypotheses were tested by means of MEG recordings of the N1m and auditory steady state response (ASSR). Both auditory evoked responses were evoked by a test tone with the same carrier frequency as the center frequency of the notch following a preceding NFN with one of various specific spectral energy contrasts. The N1m response is known to be generated in secondary auditory cortical areas [27] and studies focusing on lateral inhibitory effects of NFN in humans have mainly investigated these areas [1], [2]. However, since previous studies could demonstrate that lateral inhibition does occur in both, primary [10] and non-primary auditory cortical areas [1], [2], in addition to the N1m response we simultaneously measured ASSR, which are known to be generated in primary auditory cortices [27].

## Experiment 1

### Methods

#### Ethics statement

The study was conducted according to the Declaration of Helsinki and the study protocol was approved by the ethics committee of the Medical faculty of the University of Münster.

#### Subjects

Sixteen right-handed subjects (mean age = 27.38; SD = 3.01; 8 males) with normal hearing (<20 dB HL) in the frequency range between 250 and 8000 Hz (in steps of one octave) as evaluated by clinical audiometry and no history of otological (e.g. no tinnitus symptoms) or neurological disorders participated in the experiment. Subjects were fully informed about the study’s content and gave written consent before participating.

#### Stimuli and experimental design

Stimuli consisted of five different masking stimuli (MS) and a test stimulus (TS). The MS consisted of white noise passing through a digital notch filter centered at 1 kHz (notch depth 60 dB). A bandwidth of ¼ octave was used because it has been shown that this notch-bandwidth leads to the greatest lateral inhibitory effect [1]. All stimuli had a 0.02 s rise and fall time and were low-pass filtered at 8 kHz (due to frequency characteristics of our sound delivery system). The spectral energy of four MS was modified by amplifying or attenuating the energy in the frequency bandwidths of 3/8 or 7/8 octave on each side of the notch by 30 dB. To avoid clipping effects the amplification of EFB was achieved by attenuating the energy in all frequencies except the frequencies within the predefined EFB. To achieve the same intensity in all five MS, their total root-mean-square (RMS) values were counterbalanced at the same level.

**Figure 2:**
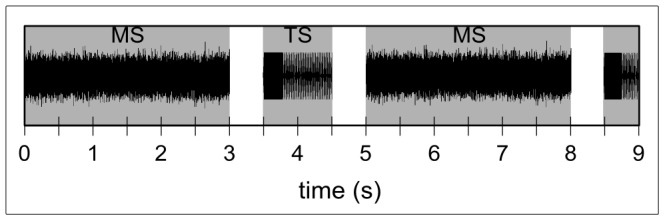
Schematic representation of a trial sequence. A masking stimulus (MS) of 3 s was followed by a test stimulus (TS) of 1 s with an inter-stimulus interval of 0.5 s.

The TS was a 1 kHz pure tone with duration of 0.3 s directly followed by a 0.7 s amplitude modulated tone (1 s total duration) with the same carrier frequency (modulation frequency of 40 Hz and modulation depth of 100%; cf. Figure 2). Presenting this stimulus type enables the measurement of transient N1m responses and sustained ASSR simultaneously [27]. Each trial consisted of a MS (duration of 3 s) and a TS (duration 1 s) with an inter-stimulus interval of 0.5 s (cf. Figure 2). The different MS were presented in a randomized order. 130 trials were presented in five runs with a short break in between each run. Each run therefore resulted in 130 trials per condition (5 conditions: 3/8 oct +30 dB, 7/8 oct +30 dB, NFN, 3/8 oct - 30 dB, 7/8 oct −30 dB; cf. Figure 1).

All stimuli were stored as digital sound files with a sample rate of 48 kHz. The sound delivery system consisted of a USB sound card (DMX 6 Fire, Terratec Electronic GmbH, Germany), a clinical speech audiometer (Midimate 622, Madsen, Denmark), a parametric 5-band equalizer (Ultra-Q Pro PEQ2200, Behringer, Germany), electrostatic ear speaker (SR-003) driven by an amplifier unit (SRM-212, Stax, Japan), and silicon plastic tubes with a length of 60 cm (inner diameter of 5 mm), which connected the earspeaker with a silicon earpiece fitted individually to each subject’s ear. To control the timing of sound presentation, the software Presentation (Neurobehavioral Systems, Albany, CA) was used. Stimulus transduction was checked by a 2 cm^3^ ear simulator (Brüel & Kjaer model 4157) that was equipped with a ½” condenser microphone (Brüel & Kjaer model 4134) and connected to the silicon earpiece at the end of the sound delivery system. Prior to each measurement, hearing thresholds for TS were determined for each ear. Sounds were presented binaurally at an intensity of 50 dB SL (above the sensation level of TS). The MS had identical power to the TS. Participants watched a silent movie during auditory stimulation to keep them alert at a more stable attentional state.

#### MEG recordings and data analysis

Auditory evoked fields were recorded in a magnetically shielded quiet room by means of a 275 channel whole-head MEG system (OMEGA 2005 WC, CTF Systems Inc., Port Coquitlam, Canada) equipped with axial gradiometer configuration of the pickup coils. Data was acquired continuously and digitally sampled at a rate of 600 Hz. Participants were sitting in a comfortable upright position. Small cotton pads stabilized the head inside the MEG dewar in order to reduce head movements. The subjects’ alertness and head position were continuously monitored during the whole experiment.

The recorded continuous data were segmented into epochs of 1.3 s with respect to the TS of 1 s duration, 0.2 s pre-stimulus and 0.1 s post-stimulus interval. A notch filter at 50 Hz and its harmonics was applied. The data epochs were baseline corrected using the pre-stimulus interval from −0.2 to −0.1 s. Epochs with field changes larger than 2.5 pT were considered as artifacts and were rejected. Subsequently, data was averaged for each condition and a grand average for all conditions was additionally calculated.

The averaged N1m responses elicited by the TS were digitally filtered with a 30 Hz low-pass filter (zero-phase, Butterworth, fourth order). Source analysis based on a single fixed equivalent current dipole (ECD) model for each hemisphere was applied to the measured field distribution of N1m responses grand averaged across the TS in all conditions. The grand averaged N1m responses, instead of the averages for each condition, were used for improving the signal-to-noise ratio. The N1m peak was defined as the maximum RMS value of the global field power across all channels in the time interval from 0.085 to 0.145 s. The 0.016 s time window prior to the N1m peak was used for the approximation of an ECD to determine the strength of a single ECD in each hemisphere. Dipole location and orientation in the left and right hemisphere were determined in a head-based Cartesian coordinate system with origin at the midpoint of the medial-lateral axis (y-axis) between the entrances of the left and right ear canals. The posterior-anterior axis (x-axis) was defined as the axis between the nasion and the origin, while the inferior-superior axis (z-axis) ran through the origin perpendicularly to the x-y-plane. Estimates of the source parameters were accepted for further evaluation only if the goodness of fit was at least 90% and the distance of the ECD to the mid-sagittal plane was greater than 3 cm. The resulting ECD template was used to calculate the maximal N1m source strength for each hemisphere and condition by means of source space projection [28]. The source waveform for each condition was statistically analyzed by means of N1m peak amplitude, which was defined as the maximum value in the time interval between 0.07 and 0.13 s post stimulus onset.

ASSR was analyzed in a similar way; however, averaged data were band-pass filtered (zero-phase, Butterworth, fourth order) between 32 and 48 Hz. After a complex demodulation at 40 Hz of the grand averaged data the maximum RMS value of the global field power across all channels was calculated. The 0.008 s time-window prior to the maximum RMS value was used for the approximation of an ECD model. The maximal source strength of ASSR for each condition was again calculated by means of source space projection [28]. After a Hilbert transformation of the ASSR source waveforms, the envelopes of the source waveforms were calculated. The mean envelope in the time window from 0.5 to 1 s after stimulus onset was used for statistical analysis of the ASSR [29], [3].

To check the resulting dipole positions, the individual dipole template’s localization for N1m and ASSR were additionally transformed into Montreal Neurological Institute (MNI) space using the information derived from normalizing the individual MRI data to MNI space. All normalized dipoles were then averaged and 95% confidence intervals for the three spatial directions were calculated. In order to visualize these results, an ellipsoid with the mean normalized dipole positions of all subjects in the center and its axis determined by the confidence boundaries was projected to a standardized MRI brain, using MRIcron software (http://www.mricro.com/mricron).

To analyze the data statistically, the maximal source strengths of N1m responses elicited by the TS were normalized with respect to the average of the maximal source strengths of all conditions in both hemispheres for each subject. This normalization procedure was applied to account for individual differences in source strengths. A repeated-measures ANOVA was applied to the normalized N1m source strengths with two factors (NFN-type: 3/8 oct +30 dB, 7/8 oct +30 dB, NFN, 3/8 oct −30 dB, 7/8 oct −30 dB; hemisphere: left and right). Before calculating each ANOVA, Mauchly’s test was conducted. If the sphericity assumption was violated, data was Greenhouse-Geisser corrected. Planned comparisons were additionally calculated by means of linear and simple contrasts for the factor NFN-type. If post-hoc comparisons were applied, they were Bonferroni corrected. All analyses were two-tailed unless otherwise specified. The same statistical analysis was applied to the ASSR source strengths.

### Results

#### N1m

**Figure 3:**
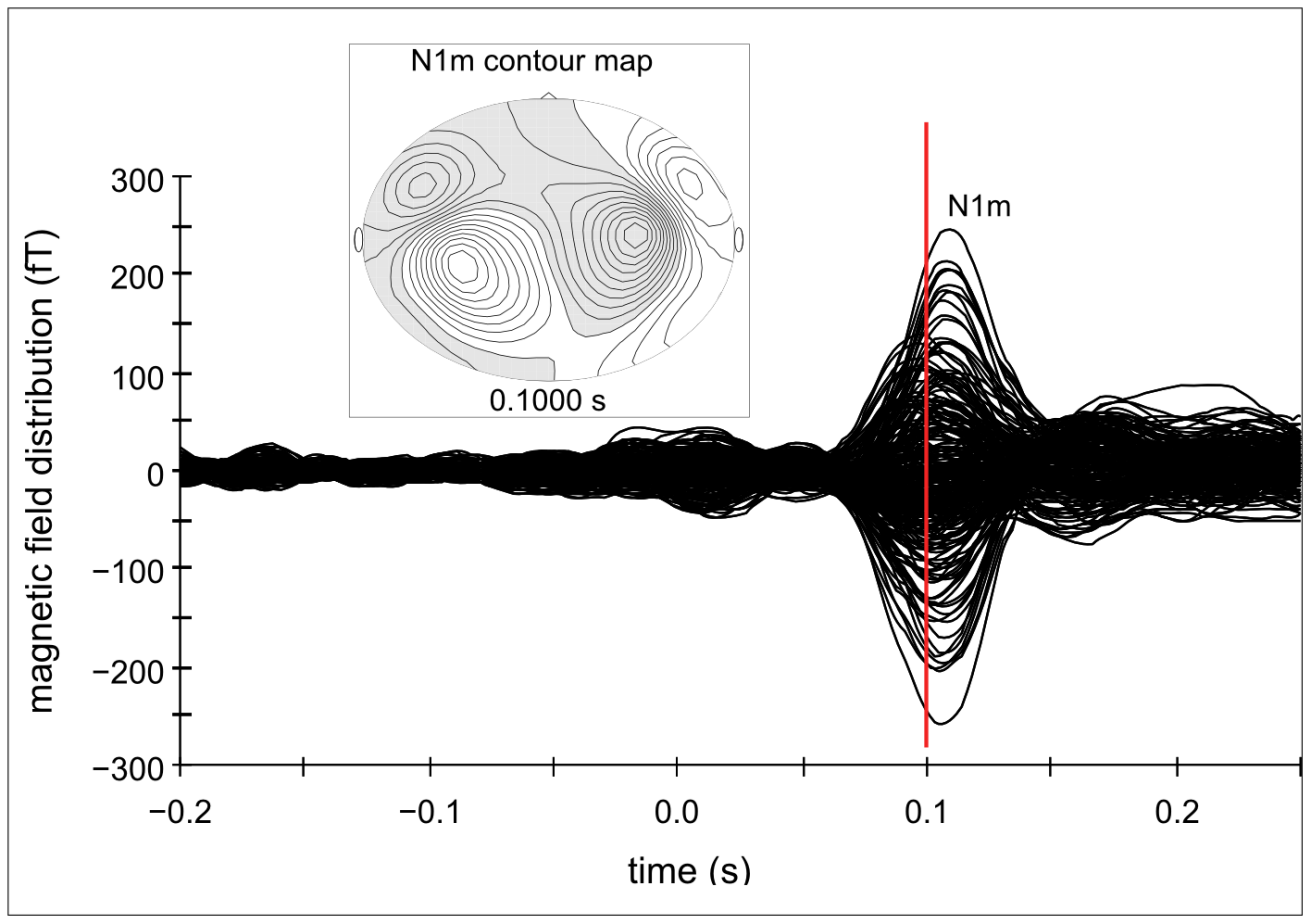
Auditory evoked magnetic field and contour map for the N1m response – experiment 1. Example of the auditory evoked magnetic field and the corresponding contour map of a representative subject for N1m responses.

**Figure 4:**
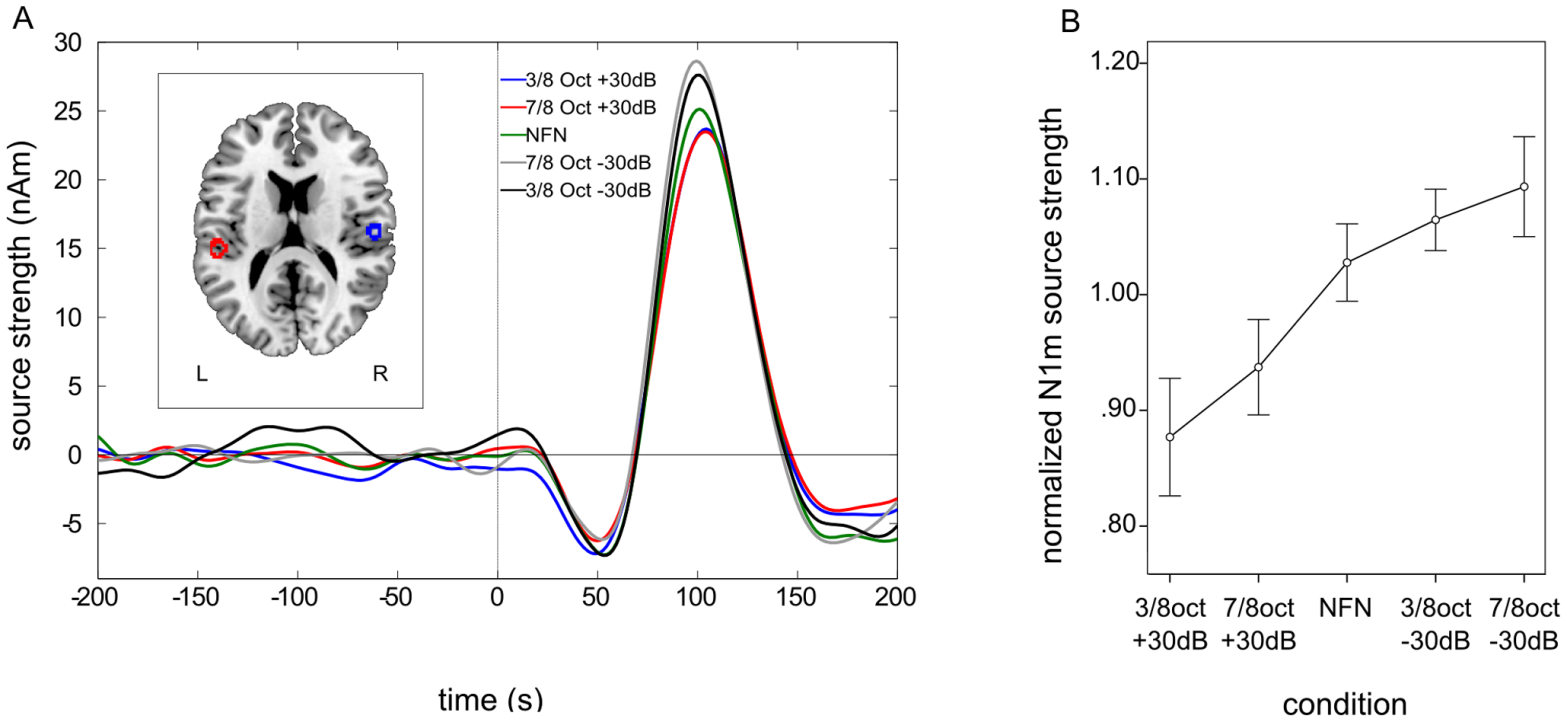
Grand averaged source waveforms, normalized source localizations and normalized N1m responses – experiment 1. A. Grand averaged source waveforms for the N1m time window in the first experiment. N1m source strength is smallest in both amplified edge frequency band (EFB) conditions and greatest in both attenuated EFB conditions. The left panel shows the normalized source locations of both equivalent current dipoles (ECD) transformed to a standardized magnetic resonance imaging (MRI) brain. B. Mean normalized N1m values demonstrating the steady decrement of N1m responses with the smallest N1m source strength in the 3/8 octave amplified condition and the greatest in the 7/8 octave attenuated condition.

Clearly identifiable N1m responses were obtained from 12 out of 16 subjects (mean age = 27.67; SD = 3.06; 6 males). Only the values of these subjects were used for further analysis. Contour maps showed a clear dipolar pattern (cf. Figure 3) and the estimated dipolar sources were located in the medial surface of the temporal lobe (cf. Figure 4 A). The grand averaged source waveforms showed a strong differentiation between the amplified and attenuated conditions compared to NFN for N1m responses, with the smallest N1m responses in both amplified conditions (3/8 oct +30 dB and 7/8 oct +30 dB) and the greatest N1m responses in both attenuated conditions (3/8 oct −30 dB and 7/8 oct −30 dB).

Repeated-measures ANOVA revealed a highly significant main effect for the factor NFN-TYPE, F (4, 44) = 4.11, *p = *0.006, η_p_^2^ = 0.27. No significant difference between hemispheres was found, F (1, 11) = 0.00, *ns*. However, there was a significant interaction effect between NFN-TYPE and hemisphere, F (4, 44) = 2.63, *p = *0.047, η_p_^2^ = 0.19.

Planned linear contrasts were applied to the normalized N1m source strengths for each condition (in the following order: 3/8 oct +30 dB, 7/8 oct +30 dB, NFN, 3/8 oct −30 dB, 7/8 oct −30 dB) which revealed a highly significant linear trend, F (1, 11) = 7.43, *p = *0.010 (one-sided), η_p_^2^ = 0.40, indicating a linear increase of N1m source strengths, with the smallest normalized N1m source strength in the 3/8 octave amplified condition (mean = 0.88, SD = 0.18) and the greatest N1m source strength in the 7/8 octave attenuated condition (mean = 1.09, SD = 0.15; cf. Figure 4 B). Simple contrasts were also calculated using the NFN-condition as a reference category. Normalized N1m source strengths in the 3/8 oct +30 dB condition were significantly smaller as compared to NFN condition (mean = 1.03; SD = 0.12), F (1, 11) = 4.02, *p = *0.035 (one-sided), η_p_^2^ = 0.27. The normalized N1m source strengths in the 7/8 oct +30 dB condition (mean = 0.94, SD = 0.14) were also smaller as compared to the NFN-condition, though this effect was only marginally significant, F (1, 11) = 2.94, *p = *0.057 (one-sided), η_p_^2^ = 0.21. Normalized N1m source strengths in both attenuated conditions (3/8 oct –30 dB: mean = 1.07, SD = 0.09; 7/8 oct −30 dB: mean = 1.09, SD = 0.15) showed no significant difference as compared to the NFN condition.

To further analyze the interaction effect between the factors NFN-type and hemisphere, post-hoc multicomparisons using Bonferroni correction were applied. There was a significant difference between the attenuated EFB conditions in the right hemisphere (*p* = 0.037), with a greater normalized N1m amplitude found in the 7/8 oct −30 dB condition (mean = 1.17, SD = 0.40) as compared to the 3/8 oct −30 dB (mean = 1.07, SD = 0.39) condition. No other comparisons revealed significant differences.

#### ASSR

**Figure 5:**
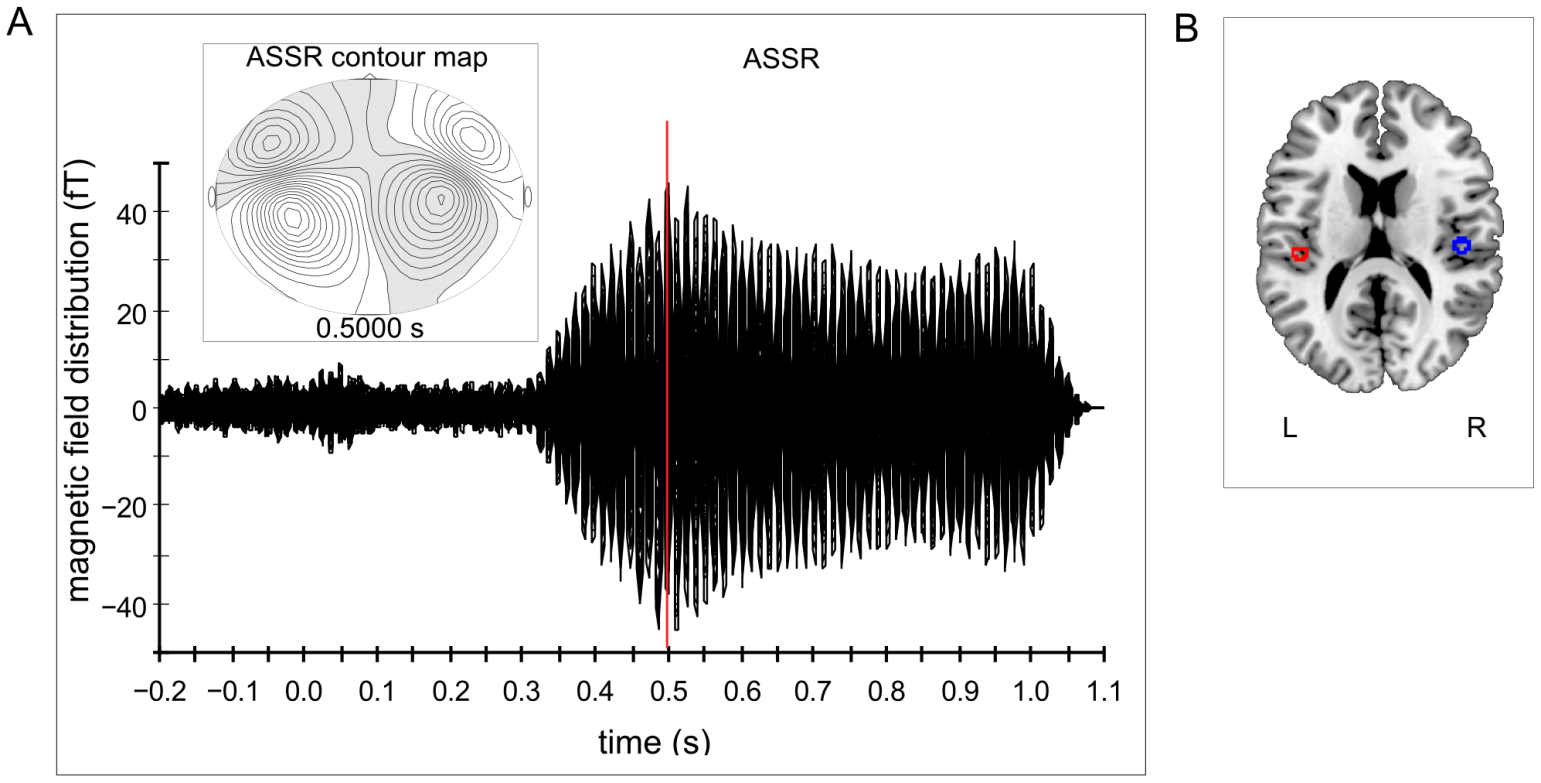
Auditory evoked magnetic field, contour map and normalized source localization for ASSR – experiment 1. A. Example of the auditory evoked magnetic field and the corresponding contour map of a representative subject for auditory steady state responses (ASSR) in the first experiment. B. Normalized source locations of both equivalent current dipoles (ECD) transformed to a standardized magnetic resonance imaging (MRI) brain.

Clearly identifiable ASSR were obtained from 14 out of 16 subjects (mean age = 26.93, SD = 2.95; 6 males). Only the values of these subjects were used for further analysis. Contour maps again showed a clear dipolar pattern and the estimated dipolar sources were located in the medial surface of the temporal lobe (cf. Figure 5).

Repeated-measures ANOVA revealed a highly significant main effect for the factor hemisphere, indicating that there was a greater overall activation in the right hemisphere (mean = 1.08, SD = 0.09) than in the left one (mean = 0.92; SD = 0.09), F (1, 13) = 12.08, *p = *0.004, η_p_^2^ = 0.48. There was neither a significant effect for NFN-type, F (4, 52) = 1.76, *ns*, nor a significant interaction between the factors NFN-type and hemisphere, F (4, 52) = 0.73, *ns*.

### Interim Discussion of Experiment 1

The source strengths of the N1m responses which were evoked by the TS clearly depended upon the spectral energy contrasts of the preceding MS. This relationship reflects lateral inhibition in the human secondary auditory cortical areas. As hypothesized, the amplification of a narrow EFB resulted in the lowest N1m source strength and the attenuation of the widest EFB led to the greatest N1m source strength. This consistent N1m decrement, which represents a steady increase in the lateral inhibition of neurons with NOTCH CF, supports the assumptions of lateral inhibitory mechanisms in auditory structures, in particular the hypothesis that excited neurons inhibit adjacent neurons more strongly than their more distant neighbors [26]. The current findings are in line with, and strongly confirm, the results of previous studies which highlighted the important role of the notch edges in NFN in the lateral inhibition of neurons with NOTCH CF [10], [1], [2], [3].

Our first hypothesis was partly confirmed, in that the amplified EFB conditions showed significant differences in N1m source strength in comparison with the reference category (NFN condition). The linear trend and mean values of the normalized N1m source strength indicate that the lowest level of lateral inhibition we measured was induced by NFN with attenuated EFB, however, a direct comparison between the NFN condition and each of the attenuated EFB conditions did not show any significant differences, when analysis included both hemispheres.

The analysis of the interaction effect between NFN-type and hemisphere revealed a significantly larger N1m source strength in the right hemisphere in response to the TS being preceded by the MS with a broad band of EFB attenuation than the MS with a narrower band of EFB attenuation. The left and right auditory cortices are functionally specialized in processing sound input: The left auditory cortex is more specialized for temporal processing and the right auditory cortex predominantly performs the spectral analysis of a sound [30], [31]. Our findings related to the attenuated EFB MS might reflect this hemispherically specialized processing, in that the right auditory cortex, which specializes in processing spectral energy contrasts, does respond significantly different to, i.e. clearly distinguishes between the two different attenuated EFB masker.

Analysis of ASSR source strength revealed significant differences between hemispheres with a greater activation in the right hemisphere, supporting a previous study [32] in which it was argued that ASSR facilitate the pitch processing of a sound based on its periodic structure. However, there was no other significant effect for ASSR source strengths. The modification of NFN with different spectral energy contrasts did not, therefore, have an influence on the primary auditory cortex, which is known to be the main generator of the ASSR, but rather on secondary auditory cortical areas, represented by the modulation of N1m source strengths.

The aim of this study was not only to investigate the effects of spectral energy contrasts on lateral inhibition in order to get new insights into the processing of sounds in the human auditory cortex. We wanted to identify further parameters, the modification of which might enhance lateral inhibitory mechanisms and therefore increase the effectiveness of tinnitus treatments such as the TMNMT [20], [21]. Experiment 1 found that N1m responses were modulated by different spectral energy contrasts, in particular that a narrow width of EFB amplification resulted in the greatest decrement of N1m source strength, reflecting the greatest lateral inhibition of neurons with NOTCH CF. Consequently, the question arises of whether an even narrower width of EFB amplification around the notch leads to yet further enhancement of lateral inhibition of neurons responsive to NOTCH CF. Therefore a second experiment was conducted in order to investigate this question in more detail.

## Experiment 2

### Introduction

In this experiment we investigated the impact upon lateral inhibition of amplified EFB with two different bandwidths, both narrower than in the first experiment, in comparison to NFN without any additional spectral contrasts. It was again hypothesized that both amplified EFB MS would induce greater lateral inhibition of neurons with NOTCH CF than the NFN condition. A consistent decrement in the amount of lateral inhibition was also assumed, with the most lateral inhibition occurring in the narrowest EFB amplification condition and the least lateral inhibition in the NFN condition.

### Methods

#### Subjects

11 of the subjects who showed clear N1m responses in the first experiment were invited to participate in the second experiment (mean age = 27.64; SD = 3.20; 5 males).

#### Stimuli, experimental design and data analysis

The stimuli consisted of a TS and three MS. The TS was the same as used in experiment 1. This time the spectral energy of the two MS was modified by amplifying the energy in the EFB of just 1/8 or 1/24 octave on each side of the notch by 30 dB. All other parameters of MS were the same as used in experiment 1. The intensity of MS was again controlled by balancing the total RMS values of all three stimuli. The experimental procedure was the same as in the first experiment, though there were only three conditions this time and therefore only three runs with 130 trials per condition (1/8 oct +30 dB, 1/24 oct +30 dB and NFN) and per run. The data analysis described in the first experiment was applied to the data acquired in the second experiment.

### Results

#### N1m

**Figure 6:**
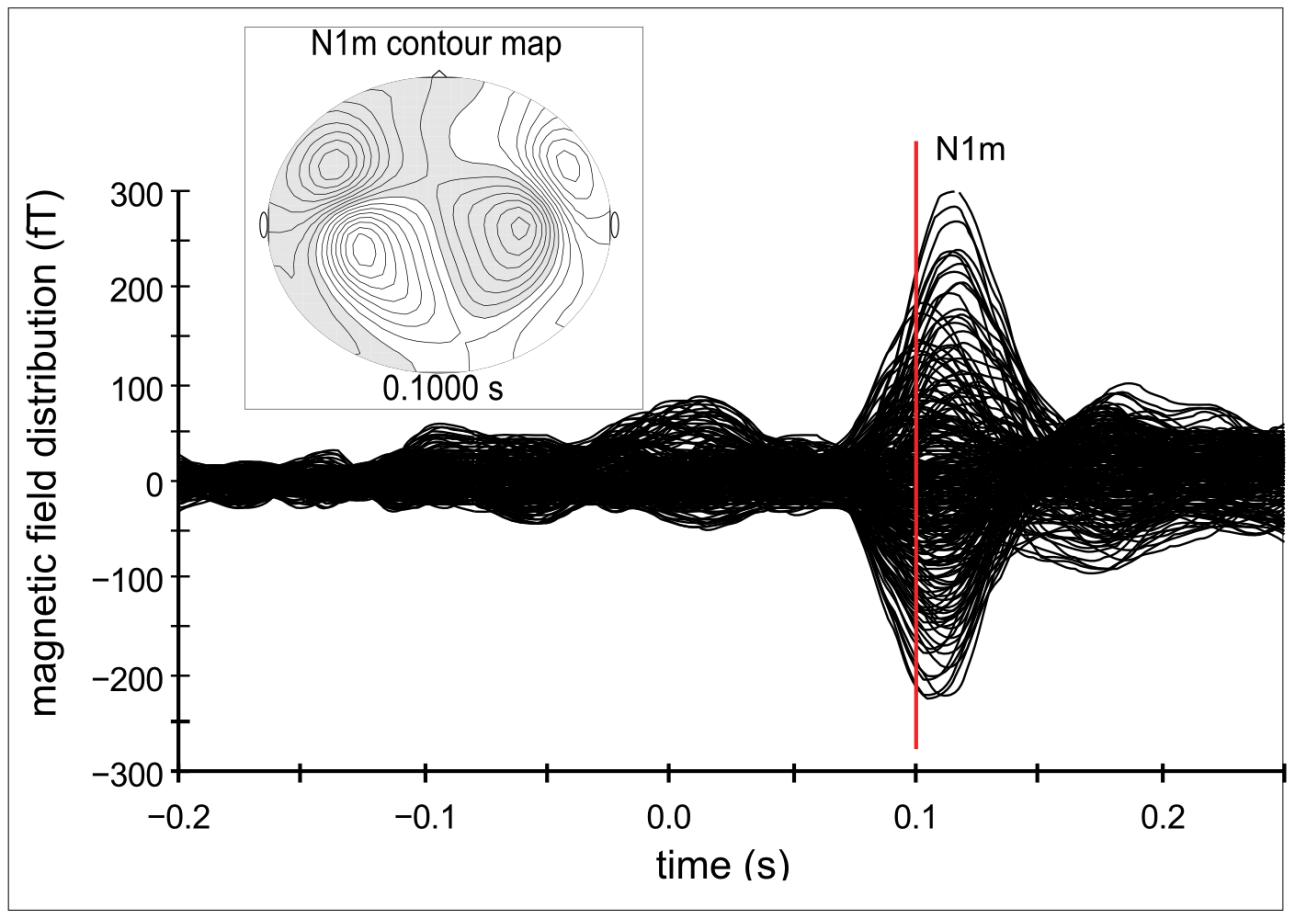
Auditory evoked magnetic field and contour map for the N1m response – experiment 2. Example of the auditory evoked magnetic field and the corresponding contour map of a representative subject for N1m responses.

**Figure 7:**
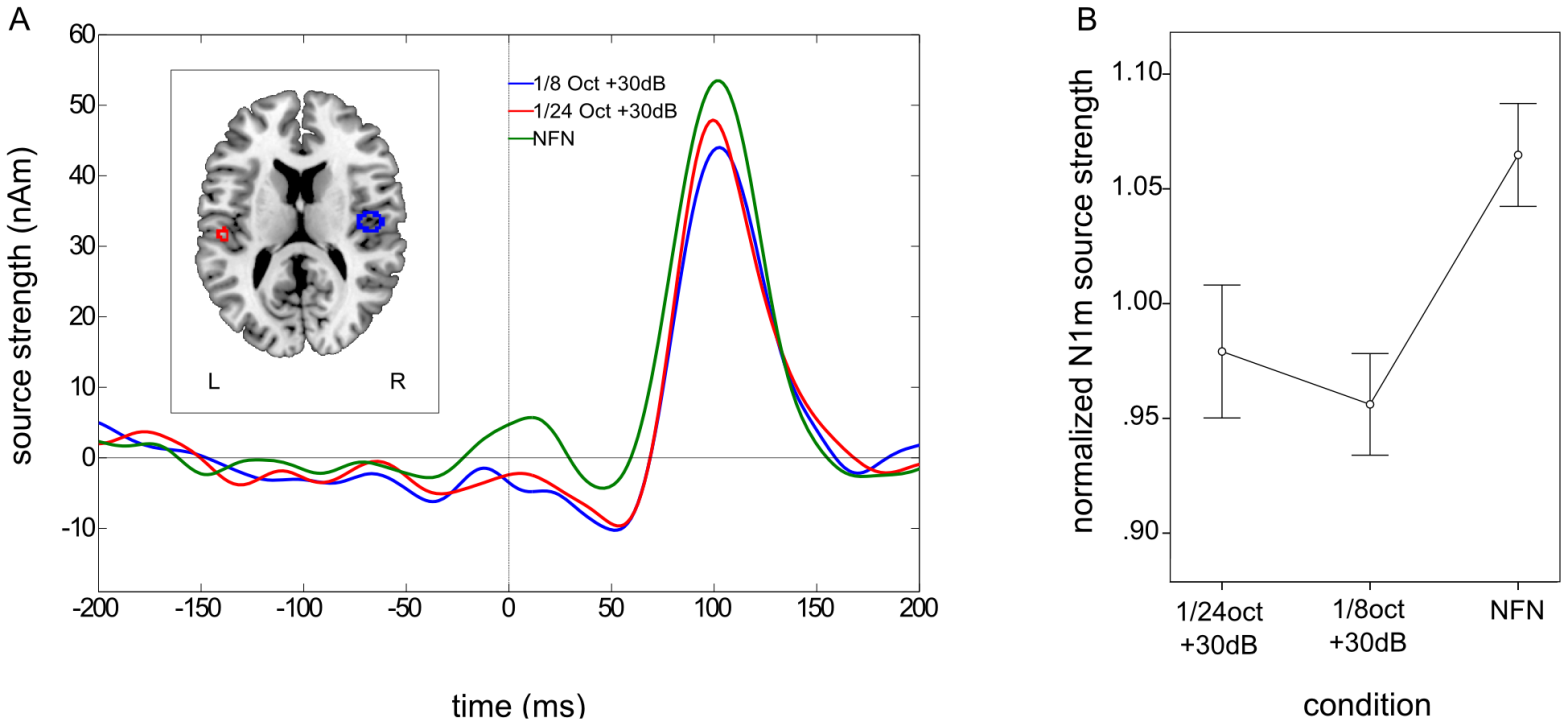
Grand averaged source waveforms and normalized N1m responses – experiment 2. A. Grand averaged source waveforms for the N1m time window in the second experiment. N1m source strength is lower in both amplified edge frequency bands (EFB) conditions than in the notch-filtered noise (NFN) condition. N1m source strength in the narrower EFB amplification condition (1/24 oct +30 dB) seems to be greater than in the wider EFB amplification condition (1/8 oct +30 dB). The left panel shows the normalized source locations of both equivalent current dipoles (ECD) transformed to a standardized magnetic resonance imaging (MRI) brain. B. Mean normalized N1m values confirming the observed results in the grand averaged source waveforms.

Clearly identifiable N1m responses were obtained from all subjects. As in experiment 1, the contour maps showed a clear dipolar pattern and the estimated dipolar sources were again located in the medial surface of the temporal lobe (cf. Figure 6). The grand averaged source waveforms showed a strong difference between the amplified and the NFN condition for N1m responses with the smallest N1m source strengths occurring in both amplified conditions. However, the amplification of the narrower EFB led to a greater normalized N1m response than the wider EFB amplification (cf. Figure 7 A).

Repeated-measures ANOVA revealed a significant main effect for the factor condition, F (2, 20) = 3.58, *p = *0.047, η_p_^2^ = 0.26. No significant difference between hemispheres was found, F (1, 10) = 0.14, *ns*. However, there was a significant interaction effect between condition and hemisphere, F (2, 20) = 4.56, *p = *0.023, η_p_^2^ = 0.31.

Planned linear contrast analysis of the normalized N1m source strengths for each condition revealed no significant linear trend, F (1, 10) = 3.35, *ns*. Simple contrast analysis with NFN condition as the reference category, however, revealed highly significant differences between the normalized N1m source strengths in the 1/8 oct +30 dB condition (mean = 0.96, SD = 0.07) and the NFN condition (mean = 1.07, SD = 0.07), F (1, 10) = 10.31, *p = *0.005 (one-sided), η_p_^2^ = 0.51. The normalized N1m source strength in the 1/24 oct +30 dB condition (mean = 0.98, SD = 0.10) was also significantly smaller than in the NFN condition, F (1, 10) = 3.35, *p = *0.049 (one-sided), η_p_^2^ = 0.25 (cf. Figure 7 B).

Post-hoc multicomparisons using Bonferroni correction were applied in order to further analyze the interaction effect between the factors hemisphere and condition. Significantly smaller N1m source strengths were found in the 1/8 oct +30 dB condition (mean = 0.89, SD = 0.21) than in the NFN condition (mean = 1.08, SD = 0.28) in the left hemisphere (*p* = 0.010). No other comparisons showed significant differences.

#### ASSR

**Figure 8:**
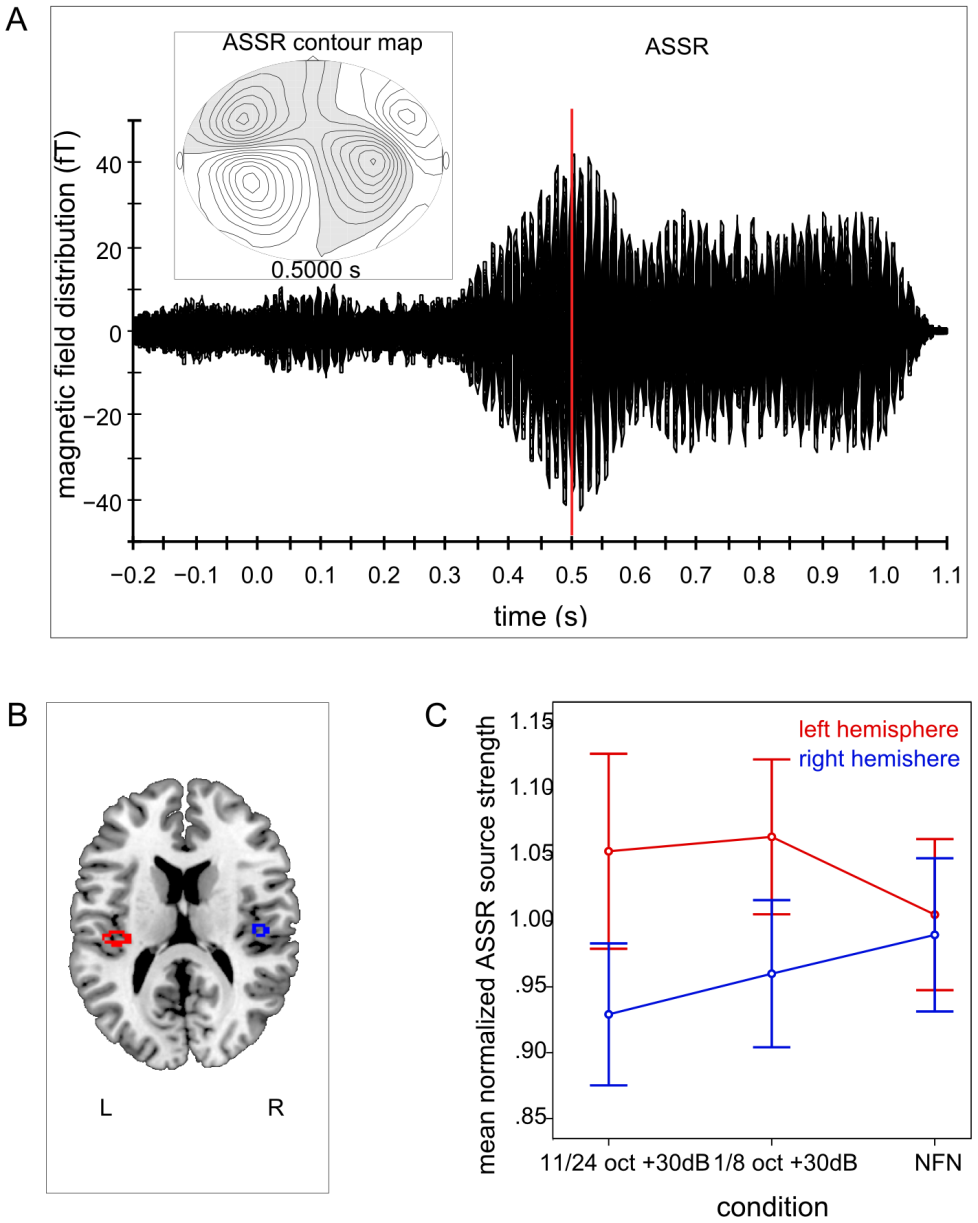
Auditory magnetic evoked field, contour map, normalized source localization and interaction for ASSR – experiment 2. A. Auditory evoked magnetic fields and the corresponding contour map of a representative subject for auditory steady state responses (ASSR) in the second experiment. B. Normalized source locations of both equivalent current dipoles (ECD) transformed to a normalized magnetic resonance imaging (MRI) brain. C. Interaction effect between the factors NFN-type and hemisphere for normalized ASSR source strengths. Error bars denote +/−1 standard error. Normalized ASSR source strengths are greater in both amplified conditions in the left hemisphere. NFN condition does not seem to differ between hemispheres.

Clearly identifiable ASSR were obtained from all subjects. Contour maps showed a dipolar pattern and the estimated dipolar sources were again located in the medial surface of the temporal lobe (cf. Figure 8).

Repeated-measures ANOVA revealed no significant main effects for the factor condition, F (2, 20) = 0.11, *ns*, or for the factor hemisphere, F (1, 10) = 0.59, *ns*. There was a significant interaction effect between the factors condition and hemisphere, F (2, 20) = 3.61, *p = *0.046, η_p_^2^ = 0.27. This interaction effect was further analyzed by applying post-hoc multicomparisons using Bonferroni correction. There was no significant difference between any comparisons. Visual analysis of the interaction (cf. Figure 8 C) revealed greater ASSR source strengths in the left hemisphere for both attenuated EFB conditions than in the right hemisphere, but ASSR source strength in the NFN condition did not appear to differ between hemispheres.

### Interim Discussion of Experiment 2

Further narrowing the width of the amplified EFB resulted in further reduced N1m source strengths, in comparison with the NFN condition, representing further enhanced lateral inhibition of neurons with NOTCH CF. This finding supports the results of the first experiment in revealing a robust effect of EFB amplification on the lateral inhibition of neurons with NOTCH CF.

The normalized mean N1m source strengths did not, however, follow a significant linear trend. We found instead a saturation effect limiting the enhancement of lateral inhibition by narrowing the width of EFB amplification. While both amplified conditions resulted in lower N1m source strengths than the NFN condition, reduction of the width of the amplified EFB beyond 1/8 octave provided no further increase in lateral inhibition. This finding might be interpreted in the way that a minimum number of excited neurons is required to induce lateral inhibition. Further reduction of the amplified bandwidth beyond 1/8 octave activates not enough neurons to provide further enhancement in lateral inhibition.

There was, in addition, an interaction effect between hemispheres and NFN-type, in that a significantly lower N1m source strength was measured in the left hemisphere in response to the broader width of EFB amplification than in the NFN condition. The first experiment demonstrated a significant difference between the attenuated EFB conditions in the right hemisphere, and this was suggested to be a consequence of spectral analysis being predominantly performed in the right hemisphere. A similar idea of specialized processing might be at work in the second experiment, in that the two MS with amplified EFB were effectively narrow-band noise signals within a broad band noise, and this could have resulted in a signal-in-noise processing, a function which is known to be pronounced in the left hemisphere [33]. Lateral inhibition of neurons with NOTCH CF could therefore have been more effective in the left hemisphere for this more narrowly amplified EFB.

The ASSR was again not affected by different spectral energy contrasts. A significant interaction between NFN-type and hemisphere was found, however this effect vanished when applying post-hoc multicomparisons. Though comparing the normalized mean source strengths of ASSR in each condition and hemisphere visually, the amplified conditions seemed to have greater source strengths in the left hemisphere than in the right one, while the NFN condition did not seem to differ between hemispheres (cf. Figure 8 C). This result might again be interpreted as an effect of signal-in-noise processing being more pronounced in the left hemisphere [33], which would be in line with the N1m results described above. The noise in the NFN condition did not have an additional narrow-band noise within the broadband noise as in both amplified conditions. This might be one explanation for the different source strengths between hemispheres for both amplified conditions and the absence of these differences in the NFN condition.

## General Discussion

The modulatory effects of spectral energy contrasts on lateral inhibition in the human auditory cortex were shown in the secondary auditory cortical areas by means of variations of the N1m response. The first experiment revealed a clear linear pattern of N1m responses evoked by the TS, with the lowest N1m source strength found in the narrow EFB amplification condition and the greatest N1m source strength found in the broad EFB attenuation condition. The second experiment revealed smaller N1m amplitudes in both amplified EFB conditions than in the NFN condition, reflecting enhanced lateral inhibition of neurons with NOTCH CF when NFN with amplified EFB is presented.

These results support previous studies which investigated the influence of spectral energy contrasts on lateral inhibition of neurons with NOTCH CF [10], [1], [2], [3]. We were therefore able to demonstrate the important role of the EFB in NFN for modulating lateral inhibition. In particular, we have shown that amplified EFB in NFN induce greater lateral inhibition of neurons with NOTCH CF than NFN without additional spectral contrasts. The effect of amplified EFB on lateral inhibition seems to be robust, because it was demonstrated in the first experiment and confirmed in the second one.

The second experiment also gave new insights into the effect of narrowing the width of EFB amplification. It was shown that narrowing the width of amplified EFB is subject to a saturation effect, which was discovered by the absence of a linear trend in N1m source strength. We had hypothesized that lateral inhibition of the NOTCH CF should be more greatly enhanced as the bandwidth of EFB amplification narrows; however, this effect reaches saturation when the width of EFB amplification is narrower than 1/8 octave on each side of the notch.

The modulation of lateral inhibition by means of spectral energy contrasts was found in secondary auditory cortical areas, as measured by N1m responses, not in primary auditory cortices, as measured by ASSR. A previous study which focused on lateral inhibitory mechanisms evoked by notch-filtered noise also found the primary auditory cortex to be unaffected by comb-filtered noise [3]. This absence of modulatory effects of spectral energy contrasts on lateral inhibition in the human primary auditory cortex was discussed in the above study in terms of the hierarchical structure of the auditory cortex, according to which primary auditory cortices process pure tones, whereas non-primary auditory cortices predominantly process more complex sounds. An fMRI study also revealed this hierarchy of sound processing [34], and our findings are in line with this model.

However, the assumption that only pure tones are processed in the primary auditory cortex has been challenged. One study demonstrated that neurons in the primary auditory cortex also respond to complex sounds [35]. Additionally, it was found that listening to notched music for 12 months (2 hours per day) resulted in a significant reduction of ASSR source strength as evoked by a TS with the same carrier frequency as the notch center frequency [20], again indicating that complex sounds, such as music, are processed in the primary auditory cortex.

Another possible explanation for the absence of any modulatory effect of spectral energy contrasts on lateral inhibition as measured by ASSR in our study might have to do with the timing of the amplitude modulated tone evoking the ASSR. Because the TS consisted of a pure tone in the first 0.3 s and an amplitude modulated tone in the last 0.7 s, the ASSR was always evoked 0.8 s (0.5 s inter-stimulus interval +0.3 s pure tone) after the masker-offset, while the N1m was always evoked 0.5 s after masker-offset. The effect of lateral inhibition is known to diminish with an increasing interval between masker and test tone, with the most effective inter-stimulus interval being 0.5 s [36]. Consequently, the effects of lateral inhibition might have only been induced for a brief period, not long enough to affect the ASSR. Further research is required to validate this assumption. One might use two TS, a pure tone and an amplitude modulated tone, instead of a successive combination of both types of signals within one stimulus. This would ensure the same inter-stimulus interval between MS and pure tone as well as between MS and amplitude modulated tone.

In this study we investigated lateral inhibitory mechanisms in the human auditory cortex and identified further modifiable parameters which might improve tinnitus treatments such as TMNMT [37], [21]. As mentioned above, an amplification of the EFB in NFN leads to the enhanced lateral inhibition of neurons with NOTCH CF. It would therefore be reasonable to expect an improvement in the effects of TMNMT on tinnitus-related neural activity and tinnitus loudness if an amplification of the EFB was applied to the notched music. It should be noted, however, that bandwidths smaller than 3/8 octave on each side of the notch might lead to an additional sound perception within the noise. This could be a disturbing factor while listening to notched music and, since the enjoyment of music plays an important role in cortical network reorganization [38], risks reducing the potential benefit of the treatment itself. On the basis of the present findings, we recommend the EFB amplification of 3/8 octave on each side of the notch as an optimal parameter. In the present study, this spectral energy contrast modification of noise resulted in a reduction of N1m source strengths by 14.5% as compared to NFN with no additional spectral energy contrasts and did not generate any additional signal-in-noise perception.

## Conclusions

This is, to our best knowledge, the first study demonstrating that the lateral inhibition of neurons with NOTCH CF in the human secondary auditory cortical areas is not only modulated by the bandwidth and transition steepness of the notch, but also by the energy and width of EFB around the notch. In particular, a purposeful enhancement of lateral inhibition can be achieved by amplifying the EFB around the notch.

These new insights can be directly transferred to tinnitus treatments which focus on the enhancement of lateral inhibition of the tinnitus frequency, such as TNMMT, by introducing amplified EFB around the notch centered at the tinnitus frequency.
