# Correction: Modulatory Effects of Spectral Energy Contrasts on Lateral Inhibition in the Human Auditory Cortex: An MEG Study

**DOI:** 10.1371/journal.pone.0104051

**Published:** 2014-07-24

**Authors:** The PLOS ONE Staff

In the third paragraph of the Results section for Experiment 1, the linear contrast is incorrectly reported as one-sided. Therefore, the correct results read as follows: “F (1, 11)  =  7.43, *p*  =  0.020, η_p_^2^  =  0.40.”

All simple contrasts are described incorrectly as F-values, though they should be t-values.

Figure 4 is incorrect. The x-axis of Figure 4A should read “time (ms)” instead of “time (s).” The authors have provided a corrected version here.

**Figure 4:**
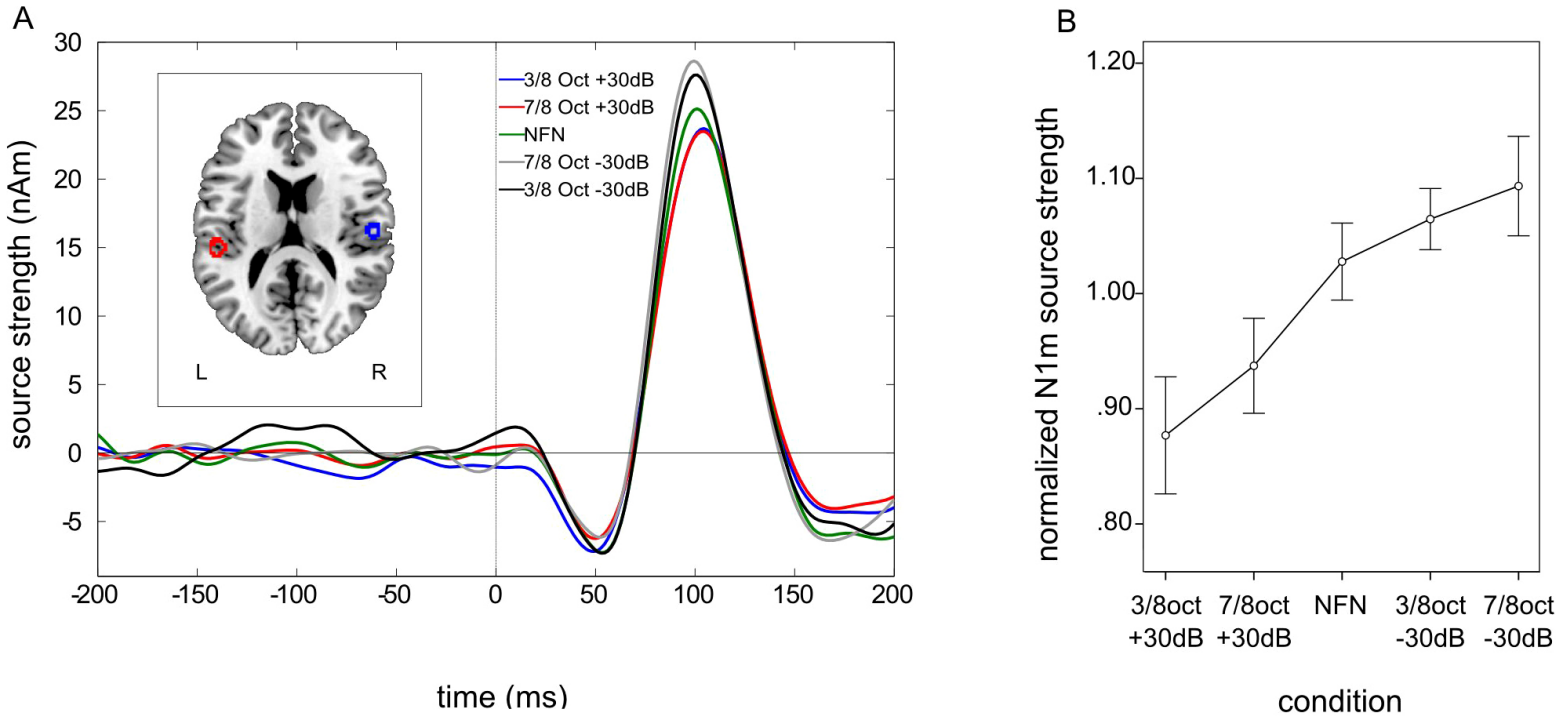
Grand averaged source waveforms, normalized source localizations and normalized N1m responses – experiment 1. A. Grand averaged source waveforms for the N1m time window in the first experiment. N1m source strength is smallest in both amplified edge frequency band (EFB) conditions and greatest in both attenuated EFB conditions. The left panel shows the normalized source locations of both equivalent current dipoles (ECD) transformed to a standardized magnetic resonance imaging (MRI) brain. B. Mean normalized N1m values demonstrating the steady decrement of N1m responses with the smallest N1m source strength in the 3/8 octave amplified condition and the greatest in the 7/8 octave attenuated condition.
